# Biotic and abiotic factors affecting soil microbial carbon use efficiency

**DOI:** 10.3389/fpls.2024.1445230

**Published:** 2024-11-15

**Authors:** Xinyu Tang, Zhenxin Li, Jihong Yuan, Weirui Yu, Wenbo Luo

**Affiliations:** ^1^ State Environmental Protection Key Laboratory of Wetland Ecology and Vegetation Restoration, School of Environment, Northeast Normal University, Changchun, China; ^2^ Key Laboratory of Vegetation Ecology, Ministry of Education, Northeast Normal University, Changchun, China; ^3^ Wetland Ecological Resources Research Center, Jiangxi Academy of Forestry, Nanchang, Jiangxi, China

**Keywords:** carbon use efficiency, soil microorganisms, biotic factors, abiotic factors, carbon cycle

## Abstract

Soil microbial carbon use efficiency (CUE) refers to the efficiency of microorganisms in converting absorbed carbon into their own biomass carbon. Soil microbial CUE is a key parameter to understanding the soil carbon cycle. Biotic and abiotic factors are widely considered to be important factors influencing CUE. However, the related underlying mechanisms remain unclear. This review elaborates on the concept of soil microbial CUE and the various approaches used for its measurement. We reviewed the effects of various abiotic factors, such as temperature, soil moisture, pH, nutrient addition, and substrate type, and biotic factors, such as microbial community structure and diversity, on CUE. Finally, we discussed the focus areas that future studies need to further explore. We hope this review can provide a comprehensive understanding of the factors impacting soil microbial CUE, which is a fundamental step to improving soil carbon storage capacity.

## 1 Introduction

Carbon cycling is one of the key biogeochemical cycling processes in terrestrial ecosystems (Schimel and Schaeffer, 2012). During this cycle, atmospheric carbon dioxide (CO_2_) is absorbed and fixed through several processes, including biological, geological, and anthropogenic disturbance processes (Zhang W. et al., 2024). Generally, terrestrial ecosystems are crucial to reducing atmospheric CO_2_ levels (Davidson and Janssens, 2006). Exploring the mechanisms of carbon cycling is fundamental to countering global climate change (Sun and Chen, 2024). Moreover, the soil carbon pool is the biggest carbon pool in terrestrial ecosystems, with carbon levels ~3- and 2-fold higher than those in the atmosphere and the plants, respectively (Soong et al., 2020). Thus, changes in the soil carbon pool might profoundly impact global carbon cycling (Lou et al., 2024). Soil microorganisms play an indispensable role in terrestrial ecosystem carbon cycling (Frey et al., 2013), participating in nearly all soil transformations, connecting soil, biosphere, atmosphere, hydrosphere, and lithosphere fluxes (Chu et al., 2017).

**Figure 1:**
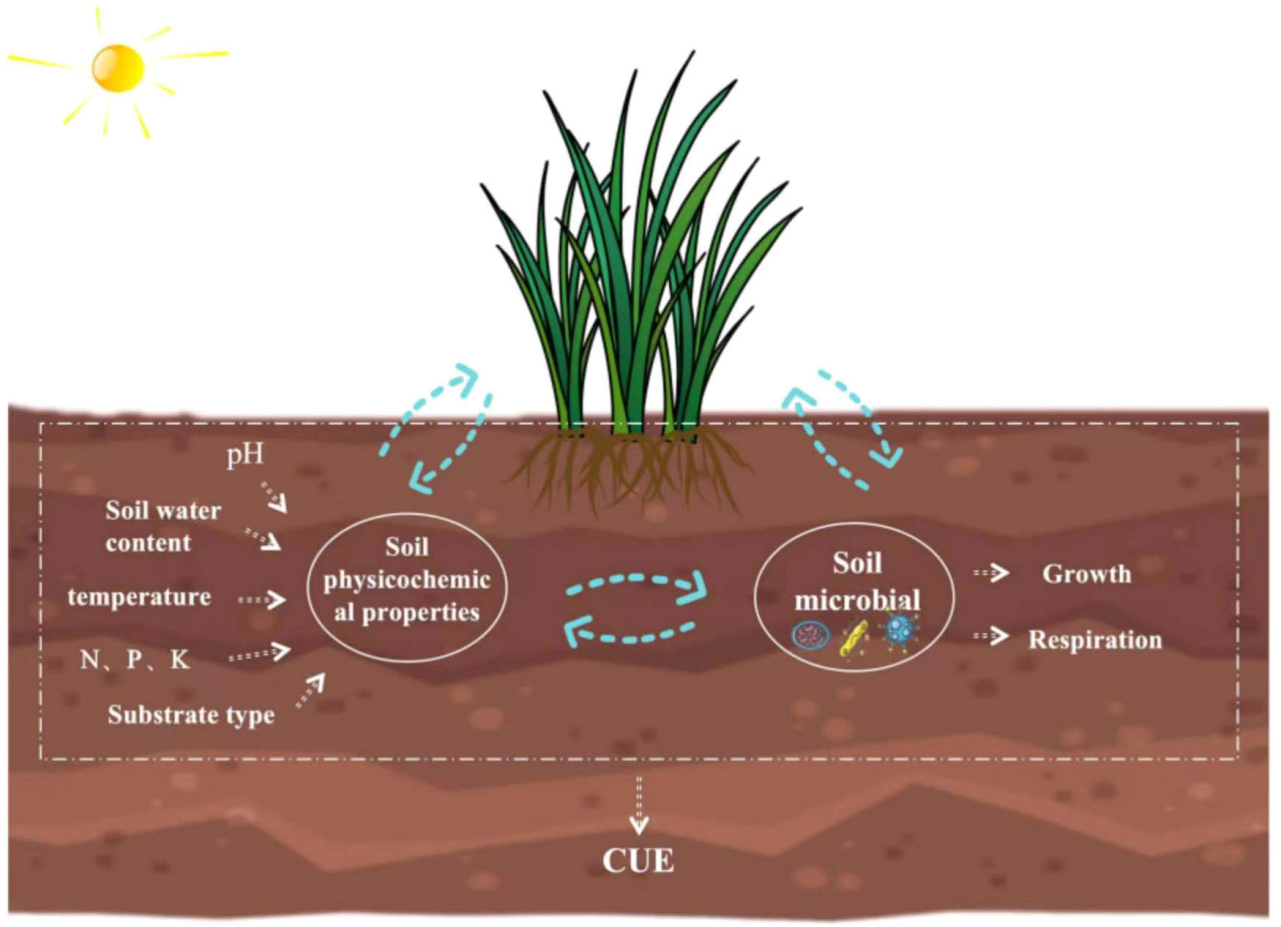
Main factors affecting soil microbial carbon use efficiency (CUE).

The carbon use efficiency (CUE) of soil microorganisms is defined as their ability to convert the absorbed carbon into biomass (Chen and Yu, 2020), which might directly influence the ecosystem carbon storage rate and storage capacity (Wieder et al., 2013; Miltner et al., 2012). Therefore, exploring soil microbial CUE is important for better understanding ecosystem carbon allocation patterns, carbon flux changes, and carbon storage status and accurately predicting the effects of global climate change on carbon cycling (Xu et al., 2014). Microbial CUE has always been depicted as a fixed variable in many soil carbon cycling models (Parton et al., 1987; Hansen et al., 1991; Kätterer and Andrén, 2001). However, several studies have found that biotic and abiotic factors influence the CUE (Adu and Oades, 1978; Song et al., 2012), such as soil moisture and water effectiveness (Tiemann and Billings, 2011), temperature (Apple et al., 2006; Wetterstedt and Ågren, 2011), pH (Malik et al., 2018; Silva-Sánchez et al., 2019), and nutrients (Ågren et al., 2001; Manzoni et al., 2012). However, the mechanisms underlying the impact of these environmental factors of soil microbial CUE remain unclear (Malik et al., 2020; Manzoni et al., 2012). For instance, some studies have shown that the CUE decreases with increasing temperature (Allison et al., 2010; Devêvre and Horwáth, 2000) because rising temperature leads to elevated respiration and subsequent substrate depletion and nutrient limitation, reducing the CUE (López-Urrutia and Morán, 2007; Allison et al., 2010). In contrast, some studies have shown that increasing temperature only mildly impacts the CUE (Hagerty et al., 2014; Tucker et al., 2013; Dijkstra et al., 2011b; Hagerty et al., 2014). Furthermore, Spohn et al. (2016b) reported a decrease in CUE with increasing nitrogen levels. However, Liu et al. (2018) found that CUE increased with rising nitrogen levels. Thus, the degrees of impact of different environmental factors on soil microbial CUE and the related underlying mechanisms are still not fully understood (**Figure 1**) (Jones et al., 2018).

## 2 Ecological concepts of microbial CUE

The term “growth yield” refers to the efficiency of an organism to convert substrate to biomass. This term was coined in the early 20^th^ century as a physiological indicator to assess the efficiency of bacteria to assimilate substrate (Monod, 1949). In the mid-1990s, Gifford introduced the term “growth yield” into the field of Ecology as a way to characterize the potential carbon sequestration capacity of organisms, but termed it Carbon Use Efficiency (CUE) (Gifford, 1995). Since then, CUE has gradually been classified into plant CUE, microbial CUE, and ecosystem CUE, referring to plant carbon assimilation, soil carbon sequestration, and ecosystem carbon use efficiencies, respectively (Manzoni et al., 2012). Of these, microbial CUE is the ratio of the amount of organic carbon substrate used by microorganisms for growth and assimilation to the amount of substrate carbon used during decomposition and alienation (Sinsabaugh et al., 2017), reflecting the partitioning of carbon between the growth and respiration of the microorganisms (Sinsabaugh et al., 2013).

In ecological research, microbial CUE is usually expressed as the ratio of microbial growth (µ) to absorption (U), that is, $CUE=\frac{\mu }{U}$ (Manzoni et al., 2012). Microbial CUE also reflects the several processes affecting carbon metabolism at different temporal and spatial scales, such as physiological processes at individual cell and species levels or kinetic features at the community and ecosystem levels (Geyer et al., 2016). Thus, it directly influences the carbon retention time, turnover rate, and soil carbon storage capacity of the ecosystem (Tucker et al., 2013). In summary, exploring the responses of soil microbial CUE to environmental factors can help assess the potential of carbon storage and accurately predict the impacts of global changes, anthropogenic disturbances, and the related management measures on carbon sequestration in ecosystems, making it a hotspot in current ecological research.

## 3 Determination of soil microbial CUE

Several methods have been used to estimate soil microbial CUE, such as the calorimetric ratio method, ^13^C-glucose tracer method, metabolic flux analysis method, ^18^O-water labeling method, and stoichiometric modeling (Manzoni et al., 2012) (**Table 1**). In 2004, Hansen et al. found that calorimetric respirometry uses the ratio of heat production to respiration to measure the CUE (Hansen et al., 2004; Spohn et al., 2016a). Calorimetric methods have poor universal applicability (Chakrawal et al., 2020) primarily because they require some empirical coefficients for CUE measurement, such as microbial biomass and oxidation state of substrate carbon. However, the range of such empirical coefficients is often limited, leading to a high CUE (Geyer et al., 2019). In 2006, while examining the differences in the growth yield efficiencies of forest ecosystems, Brant et al. suggested that ^13^C-glucose tracing might trace the uptake and mineralization of ^13^C-labeled substrates, where growth is inferred from ^13^C-incorporation into microbial biomass (Brant et al., 2006). However, this method leads to high CUE values because it uses biologically easily available carbon, inaccurately reflecting the real substrate utilization by microorganisms in the environment (Chen and Yu, 2020). In 2011, Dijkstra et al. proposed that metabolic flux analysis could be used to measure CO_2_ production for individual C atoms using position-specific ^13^C-labeled substrates (Dijkstra et al., 2011a; Spohn et al., 2016a). Metabolic flux analysis is a relatively simple method but has a low specificity, making it unsuitable for measuring the impacts of microbial turnover and secretions (Dijkstra et al., 2015). In 2016, Spohn et al. used a novel substrate-independent method of CUE assessment based on the incorporation of ^18^O from labeled water into the microbial DNA. They measured CUE by combining the conversion factors of microbial DNA and carbon biomass (Spohn et al., 2016a) to study the effects of long-term fertilization on microbial CUE and microbial biomass turnover time. However, they also showed that this method is suitable for assessing CUE in short-term culture treatments but could not be used to estimate the CUE over a long-term period (Spohn et al., 2016a). Furthermore, Sinsabaugh et al. (2016) suggested that CUE could be calculated via the activities of several soil enzymes, such as microbial extracellular enzyme activity and the carbon-to-nitrogen ratio of microbial biomass. They called this method the “stoichiometric modeling method” (Sinsabaugh et al., 2016). This method is easy but cannot reflect the actual microbial metabolic processes, and thus, has always been a surrogate indicator of CUE (Sinsabaugh et al., 2016).

**Table 1:** Comparison of different methods used for determining soil microbial CUE.

Method	Equation	Feature	Advantages and disadvantages	References
Calorespirometry	$\frac{R_{q}}{R_{co_{2}}}=469\left(1\;-\;\frac{r_{s}}{4}\right)-115\left(r_{s}-\;r_{MB}\right)\times \left[\frac{CUE}{1-CUE}\right]$	1. The marker substrate ^18^O-H_2_O needs to be added. 2. Anaerobic respiration is not considered. 3. CUE is calculated indirectly via the ratio of heat rate to respiration rate.	With much experience coefficient, the applicability is poor.	Geyer et al. (2019)
^13^C-glucose tracing	$CUE=\frac{13_{MBC}}{13_{MBC}+R}$	1. An organic tracer of ^13^C-glucose is required. 2. It is assumed that glucose metabolism is equal to microbial soil organic carbon metabolism. 3. The substrate absorption rate will decrease with time, leading to a CUE decrease with time. 4. Substrate addition might cause the priming effect, which might cause the measured CUE value to be larger than the actual value.	It is easy to operate and economical; however, the type of tracer used has an impact on the results, and the measured CUE value will fluctuate with time.	Geyer et al. (2019)
Metabolic flux analysis	$CUE=\frac{6\times V_{1}-\sum \;CO_{2}}{6\times V_{1}}$	1. A tracer with a ^13^C-labeled substrate is required. 2. It is assumed that glucose metabolism is equal to soil organic carbon metabolism. 3. Loss of carbon in the form of CO_2_ is represented by various dehydrogenase metabolic processes. 4. Anaerobic respiration is considered.	It is easy to operate and affordable, but the results are not targeted.	Dijkstra et al. (2015)
^18^O-water tracing	$CUE=\frac{18_{MBC}}{18_{MBC}+R}$	1. No organic tracer is required. 2. It is assumed that water is the only source of oxygen needed for growth. 3. It is assumed that the new cells have the same amount of DNA as the mature cells. 4. Since no organic tracer is added, the substrate absorption rate is stable, and CUE changes little over time.	CUE can be measured directly, but this method is mainly suitable for short-term studies.	Spohn et al. (2016a); Spohn et al. (2016b)
Stoichiometric modeling	$\;CUE_{C:X}=\;CUE_{max}\times \frac{S_{C:X}}{\;\;S_{C:X}+K_{X}}$	1. A tracer is not required. 2. Modeling was based on the differences in the compositions of carbon, nitrogen, and phosphorus elements in microbial biomass and respiratory substrates.	It is simple but highly dependent on model assumptions.	(Sinsabaugh et al., 2013)

Taken together, each of these methods has its own set of benefits, drawbacks, and application range (Geyer et al., 2019), warranting the need for a fast, convenient, low-cost, and more accurate CUE assessment method.

## 4 Environmental factors

### 4.1 Abiotic factors

#### 4.1.1 Temperature

Temperature is an important environmental factor affecting soil microbial CUE. Several studies have reported significantly negative effects of temperature on CUE (Frey et al., 2013; Qiao et al., 2019). Within a certain temperature range, microbial respiration intensity increases with the rising temperature, causing elevated substrate consumption, nutrient limitation and reduced soil microbial CUE (Kirschbaum, 2004; Li et al., 2019). However, other studies have shown that a rise in temperature only mildly impacted soil microbial CUE (Dijkstra et al., 2011b; Hagerty et al., 2014). In addition, the effects of temperature on soil microbial CUE might depend on the period of the experiments. For instance, Frey et al. (2013) found that short-term temperature increases might produce high levels of microbially derived carbon, leading to a rise in soil microbial CUE. When the temperature rises from 10°C to 25°C, the CUE increases by 10–40%. However, long-term temperature increases might reduce soil carbon, leading to the negative relationships between temperature increases and soil microbial CUE (Frey et al., 2013). Therefore, the precise impact of temperature change on microbial CUE remains unclear.

#### 4.1.2 Soil moisture

Soil moisture plays a very important role in soil productivity by altering the energy balance between vegetation and the atmosphere (Deng et al., 2020), another key environmental factor influencing soil microbial CUE and driving biogeochemical cycling (Tiemann and Billings, 2011). The effects of soil moisture on soil microbial CUE are complex and variable. For instance, high moisture might increase nutrient availability and promote microbial growth, altering soil microbial CUE (Domeignoz-Horta et al., 2020). Domeignoz-Horta et al. (2020) found that at high soil moisture levels (60%), microbial respiration and growth rates increased by 146% and 169%, respectively. However, the growth rate increased more rapidly than the respiration rate, resulting in an 8% increase in CUE. Whereas Tiemann and Billings (2011) showed that CUE decreased in drought conditions.

The duration of the change in the soil moisture can also significantly affect soil microbial CUE. For instance, long-term water stress reduces the solubility and absorption of soil substrates, inhibiting microbial growth (Or et al., 2007), increasing metabolic consumption, and reducing soil microbial CUE (Tiemann and Billings, 2011). In contrast, short-term water stress might stimulate a microbial response to water stress, reducing the impact of drought by increasing osmotic pressure or short-term dormancy, leading to an increased soil microbial CUE (Herron et al., 2009). Thus, the influence of soil moisture on soil microbial CUE still needs to be elucidated.

#### 4.1.3 pH

Acidic, alkaline, and neutral environments have varied impacts on soil microbial CUE. The soil microbial CUE is higher in alkaline conditions, which might be attributed to two factors (Zhang et al., 2020). Firstly, an increase in pH improves the availability of organic matter and the proportion of resources susceptible to bacterial utilization, resulting in a higher soil microbial CUE in bacteria-dominated conditions (Malik et al., 2018). Secondly, soil pH might regulate the balance between the levels of fungi and bacteria in it (F:B = 0.02–0.7), generating more bacteria and increasing microbial CUE (0.05–0.5) (Silva-Sánchez et al., 2019). In acidic conditions, soil microbes need more energy, reducing aluminum stress, and transfer more carbon to physiological processes such as respiration, leading to lower soil microbial CUE (Jones et al., 2019). However, the ability of microbial communities to store carbon might be enhanced when the soil pH is close to neutral, which is the most advantageous condition for soil organic carbon sequestration (Jones et al., 2019).

#### 4.1.4 Nutrient addition

Nutrients can alter soil microbial CUE by impacting microbial growth and biomass (Liu et al., 2018). Different types of nutrients and rate of change in their levels might affect the metabolic decomposition of soil microorganisms (Yang et al., 2024), altering microbial respiration and growth rates and soil microbial CUE (Adingo et al., 2021).

Nitrogen addition has both direct and indirect effects on soil microbial CUE (Zhang et al., 2022). The nitrogen concentration and the duration of addition directly impacts soil microbial CUE (Li et al., 2021).

For instance, short-term nitrogen additions might reduce the enzymatic metabolic cost of carbon and nitrogen acquisition by soil microorganisms, inhibit microbial respiration, and increase soil microbial CUE (Riggs et al., 2015). However, Riggs and Hobbie (2016) found that long-term nitrogen additions could lead to the gradual decomposition of active carbon pools, limiting the activities of microbial communities and substantially reducing soil microbial CUE. In addition, low and high nitrogen levels have been shown to markedly increase and decrease the soil microbial CUE by 45.12% and 27.84%, respectively (Li et al., 2021).

Furthermore, nitrogen addition also indirectly affects soil microbial CUE by impacting soil microbial biomass, diversity, and respiration (Lu et al., 2011; Cline and Zak, 2015; Liu et al., 2018). For instance, nitrogen application has been shown to significantly reduce soil microbial biomass and microbial diversity in forest and grassland ecosystems (Lu et al., 2011; Xu et al., 2024). Moreover, nitrogen application can inhibit the secretions of lignocellulosic hydrolases by saprophytic bacteria, inhibiting the ability of saprophytic microbial communities to access carbon sources (such as cellulose and hemicellulose) (Cline and Zak, 2015) and affecting soil microbial CUE. Furthermore, nitrogen addition increases the availability of soil nutrients. Hence, plants need to adjust their resource acquisition strategies, reducing the proportion of carbon allocation to the below-ground part, especially to the inter-root, causing a decrease in microbial activity, reducing excitation effects, inhibiting microbial respiration (Liu et al., 2018), and elevating soil microbial CUE. Finally, nitrogen addition might affect soil pH, leading to soil acidification, increasing the levels of activated aluminum ions, inhibiting microbial biomass and its decomposition (Riggs et al., 2015; Spohn et al., 2016b), and leading to a lower soil microbial CUE.

Several studies have shown that phosphorus addition could increase soil microbial CUE (Cleveland et al., 2002; Elser et al., 2007; Widdig et al., 2020). Phosphorus fertilization can alleviate microbial phosphorus and nitrogen limitation, increasing soil microbial CUE and reducing soil carbon loss. For example, Wang et al. (2022) confirmed that phosphorus addition increasing would alleviate microbial P and N restriction, then increased soil microbial CUE. Phosphorus addition also increases the effective phosphorus levels in the soil, leading to significantly higher microbial respiration rates and biomass, increasing the CUE (Cleveland et al., 2002). Furthermore, the duration of phosphorus addition also affects soil microbial CUE. For instance, Bååth and Anderson (2003) reported an imbalance in the stoichiometric ratio of carbon, nitrogen, and phosphorus due to long-term P addition (Khan et al., 2016), reducing soil microbial CUE. Cui et al. (2020) found that microorganisms need to produce more carbon synthase to increase phosphorus acquisition, leading to a lower CUE and reducing carbon storage in order to maintain the balanced carbon, nitrogen, and phosphorus composition required for microbial biomass homeostasis during soil phosphorus limitations.

Nitrogen and phosphorus co-addition only mildly affects soil microbial CUE (Widdig et al., 2020). This finding might be attributed to the stability of the molecular composition of soil organic carbon and the relatively minor impacts of nitrogen and phosphorus additions on the carbon composition (VandenEnden et al., 2021). Moreover, the soil characteristics, such as soil texture, also influence the soil microbial CUE (Keiblinger et al., 2010; Widdig et al., 2020).

Potassium is the most abundant inorganic cation in plant cells, playing a critical role in various plant functions, which might also affect ecosystem carbon cycling (Chen et al., 2024). However, the impacts of potassium on soil microbial CUE have been studied less than the effects of nitrogen and phosphorus. Several studies found that potassium only mildly impacts the soil microbial CUE. For instance, Spohn et al. (2016b) found that potassium levels did not impact soil microbial CUE because it is not a critical element for microorganisms. Thus, changes in potassium levels do not impact microbial carbon cycling. Onipchenko et al. (2012) also showed that potassium fertilization had insignificant effects on soil microbial CUE. In conclusion, the mechanisms underlying the impact of nutrient addition on soil microbial CUE are complex. The effects of the type and amount of the nutrients added and the duration of nutrient addition on soil microbial CUE still need to be elucidated (VandenEnden et al., 2021).

#### 4.1.5 Substrate type

Soil microbial CUE is also affected by the complexity of the substrate type (Bosatta and Ågren, 1999). Simple substrates or low molecular weight compounds are easily transported inside the cell and have less activation energy (Öquist et al., 2017). In contrast, larger or more complex molecules might undergo multiple oxidation steps to form before they are utilized (Öquist et al., 2017), potentially reducing CUE (Bosatta and Ågren, 1999; Blagodatskaya and Kuzyakov, 2008). For instance, between glucose (Jones et al., 2018) and phenol (Liu et al., 2018), using the former as a substrate leads to a higher soil microbial CUE. However, the effects of substrate type on soil microbial CUE and the underlying mechanisms are still unclear and require further study.

### 4.2 Biotic factors: microbial community structure and diversity

Both microbial community structure and diversity can affect soil microbial CUE. Interspecific variability in microbial organic matter decomposition and uptake rates (Waldrop and Firestone, 2004; Ziegler and Billings, 2011) might have varied effects on CUE (Maynard et al., 2017b, a). For instance, Adu and Oades (1978) showed that fungi exhibit a higher microbial CUE than bacteria. This finding might be attributed to the indirect impact of the biological interactions among fungi on the function of the community by influencing the community composition or increasing the rates of catabolism (Song et al., 2012; Hiscox et al., 2015). Liu et al. (2018) also found that soil microbial CUE was significantly increased with an increasing proportion of fungi in the microbial community. However, Soares and Rousk (2019) reported that soils with lower fungal-bacterial ratios (F:B) had higher microbial CUE. Therefore, the effects of microbial community structure on soil microbial CUE are still unclear.

Few studies have investigated the effects of microbial diversity on soil microbial CUE. For example, Domeignoz-Horta et al. (2020) used the gradient dilution method and showed positive relationships between microbial diversity and CUE. They also found that microbial diversity more significantly impacted the growth of soil biota than respiration (Domeignoz-Horta et al., 2020). However, the relationships between microbial diversity and CUE and the underlying mechanisms need to be further explored.

### 4.3 Combined effects

Furthermore, the combined effects of multiple environmental factors on soil microbial CUE can help in better comprehension of carbon sequestration in natural ecosystems than the effects of individual factors. It is still unclear whether biotic or abiotic factors impact soil microbial CUE more significantly. Some studies have found that abiotic factors, such as temperature (Hagerty et al., 2014), pH (Silva-Sánchez et al., 2019; Zhang et al., 2020), and soil moisture (Tiemann and Billings, 2011), might impact soil microbial CUE more prominently, whereas biotic factors only play a secondary role. For instance, Jones et al. (2019) reported pH to be a fundamental factor affecting soil microbial CUE in acidic conditions, altering CUE by regulating the proportion of fungi and bacteria. In contrast, Soares and Rousk (2019) suggested that the microbial community structure more prominently affects soil microbial CUE, whereas abiotic factors might indirectly affect soil microbial CUE by regulating microbial diversity (Fengling et al., 2018; Zhang L. et al., 2024). Thus, biotic (such as fungal-bacterial ratios) and abiotic factors (such as water and temperature) might simultaneously regulate the diversity and structure of soil microorganisms. Hence, exploring their combined effects might help better assess soil microbial CUE. The results of previous studies showed that the combined effects of environmental factors on soil microbial CUE are still unclear. Hence, the factors that prominently impact soil microbial CUE still need to be elucidated.

## 5 Future prospects

### 5.1 Addition of multiple nutrients

The growth of soil microorganisms is influenced by the combined effects of multiple nutrients, indicating that the addition of only a single nutrient might not effectively elucidate how the nutrients affect microbial growth. Moreover, the effects of the type and amount of the added nutrients and the duration of nutrient addition on soil microbial CUE are still unclear. Thus, future studies should focus on exploring the combined effects of multiple nutrients on soil microbial CUE, especially over the long term.

### 5.2 Microbial diversity

The soil microbial diversity contributes to maintaining the stability and sustainability of soil ecosystems and preventing the deterioration of the soil environment (Wertz et al., 2007). Different microbial populations exhibit varying growth and respiration rates, indicating that soil microbial diversity exhibits varying effects on soil microbial CUE. However, there is a lack of knowledge about the effects of soil microbial diversity on soil microbial CUE. Thus, the relationship between soil microbial diversity, including microbial structure and quantity, and soil microbial CUE needs to be further explored.

### 5.3 Biotic and abiotic factors

The activities of soil microorganisms are simultaneously affected by biotic and abiotic factors (McIntire and Fajardo, 2014). However, most studies only focus on the effects of individual biotic or abiotic factors on soil microbial CUE. The combined effects of biotic and abiotic factors on soil microbial CUE are still poorly understood. Exploring the combined effects of biotic and abiotic factors on soil microbial CUE might help in a more accurate prediction of the changes in soil microbial CUE with changing climate. Thus, future studies should focus on the combined effects of biotic and abiotic factors on soil microbial CUE.
